# Loss of normal Alzheimer's disease-associated Presenilin 2 function alters antiseizure medicine potency and tolerability in the 6-Hz focal seizure model

**DOI:** 10.3389/fneur.2023.1223472

**Published:** 2023-08-01

**Authors:** Leanne M. Lehmann, Melissa Barker-Haliski

**Affiliations:** Department of Pharmacy, School of Pharmacy, University of Washington, Seattle, WA, United States

**Keywords:** perampanel, levetiracetam, gabapentin, lamotrigine, valproic acid, mouse seizure model, piriform cortex, cFos

## Abstract

**Introduction:**

Patients with early-onset Alzheimer's disease (EOAD) experience seizures and subclinical epileptiform activity, which may accelerate cognitive and functional decline. Antiseizure medicines (ASMs) may be a tractable disease-modifying strategy; numerous ASMs are marketed with well-established safety. However, little information is available to guide ASM selection as few studies have rigorously quantified ASM potency and tolerability in traditional seizure models in rodents with EOAD-associated risk factors. Presenilin 2 (PSEN2) variants evoke EOAD, and these patients experience seizures. This study thus established the anticonvulsant profile of mechanistically distinct ASMs in the frontline 6-Hz limbic seizure test evoked in PSEN2-knockout (KO) mice to better inform seizure management in EOAD.

**Methods:**

The median effective dose (ED50) of prototype ASMs was quantified in the 6-Hz test in male and female PSEN2-KO and wild-type (WT) C57BL/6J mice (3–4 months old). Minimal motor impairment (MMI) was assessed to estimate a protective index (PI). Immunohistological detection of cFos established the extent to which 6-Hz stimulation activates discrete brain regions in KO vs. WT mice.

**Results:**

There were significant genotype-related differences in the potency and tolerability of several ASMs. Valproic acid and levetiracetam were significantly more potent in male KO than in WT mice. Additionally, high doses of valproic acid significantly worsened MMI in KO mice. Conversely, carbamazepine was significantly less potent in female KO vs. WT mice. In both male and female KO mice vs. WTs, perampanel and lamotrigine were equally potent. However, there were marked genotype-related shifts in PI of both carbamazepine and perampanel, with KO mice exhibiting less MMI at the highest doses tested. Gabapentin was ineffective against 6-Hz seizures in KO mice vs. WTs without MMI changes. Neuronal activation 90 min following 6-Hz stimulation was significantly increased in the posterior parietal association cortex overlying CA1 and in the piriform cortex of WT mice, while stimulation-induced increases in cFos immunoreactivity were absent in KO mice.

**Discussion:**

Acute ASM potency and tolerability in the high-throughput 6-Hz test may be significantly altered with loss of normal PSEN2 function. Seizures in discrete EOAD populations may benefit from precisely selected medicines optimized for primary ASM pharmacological mechanisms.

## 1. Introduction

Alzheimer's disease (AD) poses a pressing global health challenge due to the rapidly aging world population and the relative lack of effective disease-modifying agents. AD may also benefit from targeted personalized medicine strategies to combat disease progression. Heterogeneity permeates all aspects of the disease, from age of onset to genetic variants, and comorbid conditions, highlighting that individualized treatment strategies may be necessary. Patients with AD experience seizures at a higher rate than the general age-matched population (1, 2). Individuals with familial early-onset AD (EOAD) experience the highest risk of undetected focal seizures (3). Genetic risk factors that lead to EOAD include variations or duplications in amyloid precursor protein (APP), presenilin 1 (PSEN1), and presenilin 2 (PSEN2) genes, all of which are also associated with seizures (3–6). Preclinical studies also reinforce this heterogeneity in seizure risk associated with EOAD-related genetic variants (7–13). Despite genotype-related variability in seizure risk, uncontrolled focal seizures likely contribute to and/or worsen overall AD burden (14, 15), similar to that which arises in uncontrolled or drug-resistant epilepsy. A longitudinal study of AD patients detected subclinical epileptiform activity in 42.4% of cases; patients with seizures also had a more rapid decline in cognition and executive functioning (14). The extent of network hyperexcitability in AD has likely been vastly underestimated (14); prolonged EEG monitoring studies are infrequently conducted in individuals with AD (1, 15, 16). Foramen ovale electrode recordings detected hippocampal hyperexcitability, mesial temporal lobe seizures, and spikes in the absence of scalp EEG abnormalities or clinical manifestations (17), suggesting that seizures in AD may be easily missed and thus untreated, further accelerating functional decline in these individuals. Epileptiform abnormalities are common in AD but inconsistent in presentation across individuals (18). Nonetheless, subclinical seizures are likely a major contributor to cognitive impairments in AD as opposed to being a late-onset sequela of AD neurodegeneration (17). Limited clinical studies have assessed the benefit of selected antiseizure medicines (ASMs) administration in people with AD, although some studies are ongoing (6, 19–22). However, it is presently unknown whether mechanistically diverse ASMs may be differentially effective or tolerated in EOAD vs. the general epilepsy population, an insight that could potentially benefit intervention selection to slow the functional decline of AD.

Presenilins are intramembrane proteases that form the catalytic component of the γ-secretase enzyme. Variants in these proteins lead to the aberrant cleavage of APP to the subsequent neurotoxic Aβ1-42 hallmark of AD and accumulation of Aβ plaques (23). However, presenilin (PSEN) variants actually reduce overall proteolytic activity, thereby indirectly increasing Aβ protein aggregation (24). PSEN2 is also the predominant γ-secretase in microglia (25, 26) and worsens inflammatory response in response to stimuli (27), making it an attractive target to study non-neuronal mechanisms of AD pathology. Clinically, PSEN1 variants are much more commonly causative for AD than either PSEN2 variants or APP duplications (28, 29). Studies frequently assess how PSEN1 variants may promote AD in the setting of APP duplication mutations (6, 30, 31). Furthermore, seizure susceptibility in mouse AD models with APP duplication and PSEN1 variants has been extensively studied (7, 9, 10, 12, 32–34). However, PSEN2 is also an attractive target to interrogate the biological heterogeneity of AD risk and AD-associated comorbidities, especially considering that hyperexcitability and seizures in people with PSEN2 variants are as common within 5 years of AD diagnosis as in people with APP duplications (3). Although PSEN2 variants are fewer in number in the EOAD population, the relevance of PSEN2 function to contribute to subsequent AD pathology and pathobiology carries the potential to uncover non-neuronal mechanisms associated with AD pathogenesis and epileptiform activity (5, 25–27).

The preclinical profiling of ASM efficacy and tolerability has been historically defined in young, neurologically intact male wild-type rodents (35–37), which does not wholly reflect the extent of epilepsy prevalence across the lifespan (38). There has been a concerted effort to improve twenty-first-century ASM discovery efforts to address these remaining unmet medical needs of people with epilepsy (39, 40), including increasing the integration of syndrome-specific models of pediatric epileptic encephalopathies and models of drug-resistant epilepsy into early ASM discovery (41–43). However, this approach does not go far enough to address the pressing global increase in seizures in older adults including individuals with seizures in AD. Thus, we sought to address this preclinical gap by quantifying ASM potency and tolerability in PSEN2-knockout (KO) mice to determine whether mice with an AD-related genotype and a breeding strategy suitable for efficient high-throughput ASM discovery could reflect a useful preclinical ASM screening platform for seizures in individuals with AD. Most pathogenic PSEN2 variants in AD lead to a biochemical loss of normal γ-secretase enzyme function (24, 28). PSEN2-KO mice are therefore a reasonable surrogate to evaluate the functional impacts of evoked or chronic seizures due to their facile breeding strategy (KO x KO), longevity (13), and adaptability to high-throughput drug assessments or subsequent cognitive comorbidity evaluations (6, 11, 13). Thus, we quantified the potency of distinct pharmacological classes of ASMs that are commonly prescribed to older adults with epilepsy (38) in this rodent model with an AD-related genotype to potentially guide the selection of ASMs in the clinical management of seizures in AD. We employed the well-characterized and high-throughput evoked mouse 6-Hz seizure model of limbic seizures to address this major gap (44–47). The 6-Hz model electrically induces acute, secondarily generalized focal seizures in the rodent forebrain with a high-throughput capacity (44–47). The 6-Hz seizures engage limbic structures at higher current intensity (44), regions that are also hyperexcitable in AD (17, 18), and suitably differentiate ASMs vs. other seizure and epilepsy models [i.e., maximal electroshock test, subcutaneous pentylenetetrazol, kindling models, and status epilepticus-induced chronic epilepsy models (37, 41, 48)]. We thus hypothesized that the loss of normal PSEN2 function would alter the anticonvulsant activity profile of mechanistically distinct ASMs in this preclinical seizure model and establish the differentiation capacity of the 6-Hz seizure test evoked in PSEN2-KO mice as a suitable strategy for ASM discovery for seizures in AD.

## 2. Materials and methods

### 2.1. Animals

Male and female PSEN2-KO mice were bred at the University of Washington (UW) from stock originally acquired from the Jackson Laboratory. PSEN2-KO mice breed normally (49) and are viable for at least 14 months in our laboratory (13); therefore, breeding was between PCR-confirmed PSEN2-KO males and females. Age-matched male and female WT mice were acquired from the Jackson Laboratory at 7 weeks of age, and housed alongside PSEN2-KO mice at the UW until behavioral testing 1–2 months later. All animal studies were approved by the UW Institutional Animal Care and Use Committee (protocol 4387-01), with housing conditions previously published (50). All tests were performed during the hours of 900 and 1,700. All mice were tested between 3 and 4 months of age. Mice were used for no more than two ASM efficacy tests separated by a minimum of three stimulation-free days (48). Prior to all experimentation, mice were given a minimum of 1 h to acclimate to the procedure room. Animals were euthanized by CO_2_ asphyxiation or live decapitation after all seizure testing, as specified.

**Figure 1:**
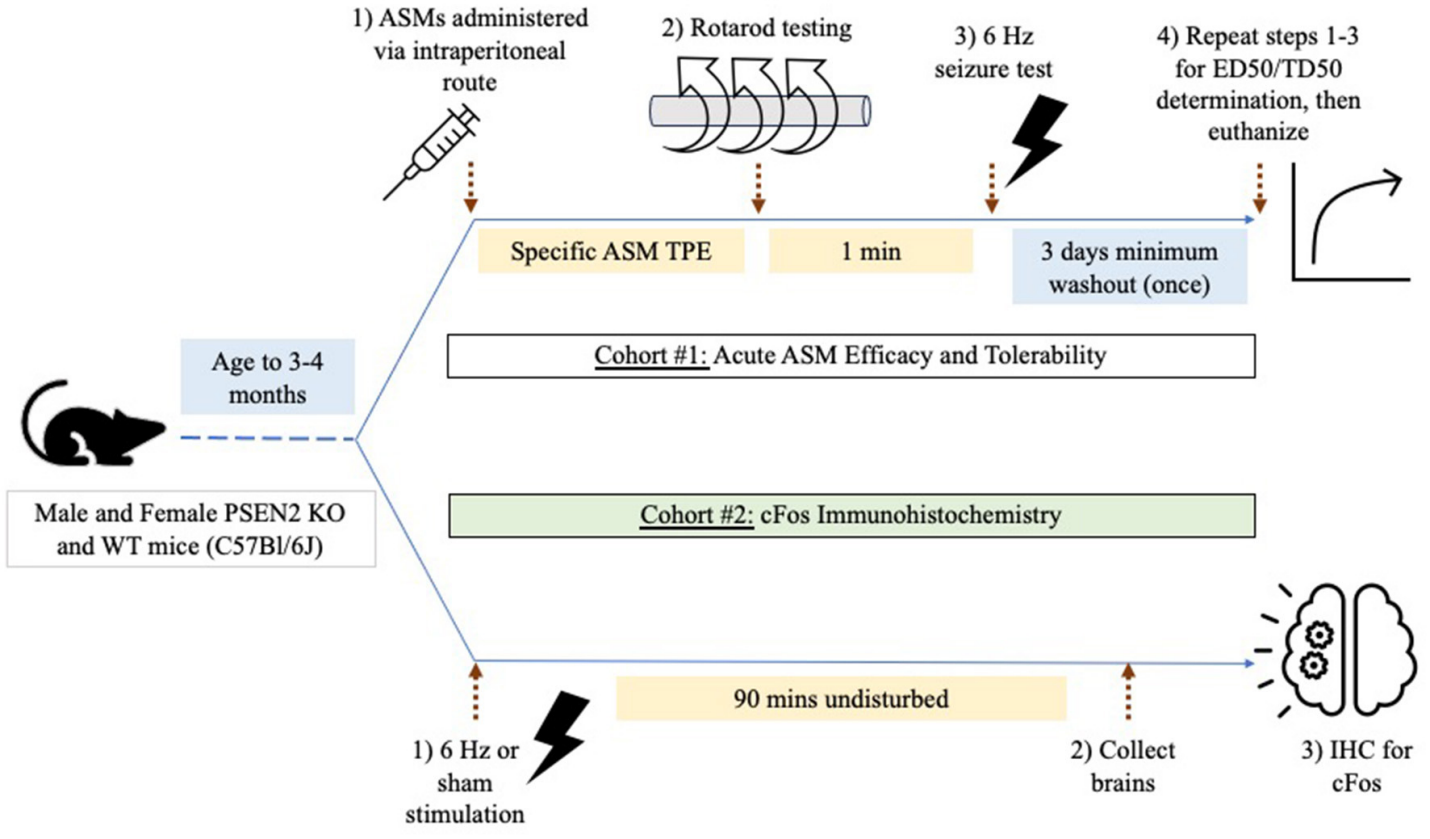
Experimental study design. Male and female PSEN2-KO and WT mice aged 3–4 months were divided into two experimental cohorts. Cohort 1 was used to assess the impact of acute administration of prototype antiseizure medicines (ASMs) administered by the intraperitoneal route and tested at the previously determined time of peak effect (TPE) for each agent. Mice were challenged on a fixed-speed (6 rpm) rotarod 1 min prior to 6-Hz seizure testing to determine a median effective (ED50) or median behaviorally impairing (TD50) dose for each ASM. Cohort 2 was used to quantify the extent of cFos immunoreactivity 90 min after a sham or 6-Hz transcorneal stimulation.

Two cohorts of mice were used for testing (Figure 1). Cohort #1 was used for ASM efficacy and tolerability testing in the 6-Hz assay (*n* = 156 female PSEN2-KO and 143 female WT mice; 128 male PSEN2-KO and 134 male WT mice). Cohort #2 was used solely for immunohistochemistry studies and did not receive ASMs (*n* = 9 female PSEN2-KO and 20 female WT mice; 10 male PSEN2-KO and 20 male WT mice).

### 2.2. 6-Hz seizure test

The 6-Hz test is considered a model of evoked secondarily generalized focal seizures that engages limbic structures at higher intensities (44). Seizures were induced by a low-frequency (6 Hz) and long-duration (3 s) stimulus delivered to anesthetized corneas through bilateral electrodes (41, 48). The evoked 6-Hz seizure is characterized by an initial momentary stun followed immediately by forelimb clonus, twitching of the vibrissae, and Straub tail (44). Animals not displaying this behavior were considered “protected.” Prior to commencing ASM studies, the median convulsive current (CC50) for both male and female PSEN2-KO mice aged 3–4 months was confirmed to be consistent with our previously reported values in male PSEN2-KO mice [i.e., 41.9 mA (95% confidence interval 39.3–46.9)] and female PSEN2-KO mice [34.4 mA (30.4–38.5); Supplementary Figure 1 (11)]. Notably, we have previously demonstrated that the 6-Hz CC50 of PSEN2-KO mice is not different from WT male and female mice at this age range, but we also confirmed that the CC50 of WT female mice [35.7 mA (30.5–39.8)] was not different from PSEN2 KO (Supplementary Figure 1). For all *in vivo* ASM testing and cFos immunohistochemistry, a 6-Hz stimulation current equivalent to the male PSEN2-KO CC95 [49.7 mA (45.3–75.6)] was used. Notably, this value was not different from the calculated CC95 in PSEN2-KO female mice [51.2 mA (43.6–89.0)].

### 2.3. Acute ASM efficacy

ASM efficacy studies in the 6-Hz test were conducted in Cohort #1 PSEN2-KO and WT mice. Seizure scores were assessed as a binary outcome of “protected” or “not protected.” ASMs were tested at their previously established time of peak anticonvulsant effect presented in Table 1 (41, 48, 51).

**Table 1:** Acute minimal motor impairment (MMI) on the fixed-speed rotarod was assessed in male and female wild-type (WT) and PSEN2-KO mice aged 3–4 months old following intraperitoneal administration of mechanistically distinct ASMs delivered at the previously determined time of peak effect for each agent.

**ASM (highest dose tested)**	**Vendor (catalog #)**	**Time of peak effect (h)**	**Female WT**	**Female WT PI**	**Female PSEN2 KO**	**Female PSEN2-KO PI**	**Male WT**	**Male WT PI**	**Male PSEN2 KO**	**Male PSEN2-KO PI**
VPA (300 mg/kg)	Sigma-Aldrich (P4543)	0.25	3/8	~2.1	1/8	>4.1	2/8	>2.2 (^†^1.7)	**6/8** ^*^	***~** **4.7***
LEV (54 mg/kg)	TCI Chemicals (L0234)	1.0	0/8	>5.4	0/8	>9.6	0/8	>2.7 (^†^18.6)	0/8	>11.3
LTG (56 mg/kg)	AK Scientific (K499)	0.5	0/8	>2.3	**3/8** ^*^	***~** **5.4***	0/8	>4.1 (^†^3.6)	**4/8** ^*^	***~** **4.2***
PER (2 mg/kg)	Cayman Chemical Co (23003)	1.0	3/8	~2.5	**0/8** ^*^	***>2.6***	5/8	~2.8	3/8	~4.9
GBP (500 mg/kg)	TCI Chemicals (G0318)	2.0	1/8	>2.0	0/8	~1.0	3/7	~5.3	1/8	~1.0
CBZ (40 mg/kg)	Sigma-Aldrich (C4024)	0.25	3/5	~5.0	1/8	>1.8	6/6	< 2.5 (^†^3.0)	**0/10** ^*^	***>2.5***

Testing in this behavioral assay immediately preceded acute transcorneal 6-Hz stimulation needed to evoke a single focal seizure. Data are presented as the number of mice impaired/the number of mice tested at each dose. Impairment proportion values in italics and bold are significantly different from sex-matched WT mice with ^*^p < 0.05. Notably, some of the ASMs tested were associated with significant genotype- or sex-related differences in MMI at the highest dose tested. An approximate protective index (PI) was also estimated for all animals based on the highest dose tested and the median effective dose of each agent (ED50).

^†^Indicates previously published PI for 6 Hz equivalent in male C57BL/6N mice from Charles River (48).

### 2.4. Minimal motor impairment

Immediately prior to ASM activity testing in the 6-Hz test, minimal motor impairment (MMI) was assessed in all mice, consistent with our prior reports (41, 48, 51). MMI was assessed using the fixed-speed rotarod (52). Mice were considered impaired if they fell 3 or more times off this rod over the course of 1 min. The extent of impairment (“impaired”/number of mice tested) at each dose was tabulated for all experimental groups. A median behaviorally impairing dose (TD50) was not calculated for any ASM. However, MMI data were used to estimate a protective index (PI; TD50/ED50) for each ASM in each sex and strain.

### 2.5. Antiseizure medicines

All ASMs were formulated in 0.5% methylcellulose (VEH; Sigma-Aldrich, M0430) and administered by the intraperitoneal (i.p.) route (Table 1), as previously described (41, 48, 51). ASMs represented distinct pharmacological classes commonly used in epilepsy and epilepsy in older adults (38, 53, 54): broad spectrum (valproic acid; VPA), sodium channel blockers (carbamazepine; CBZ; lamotrigine; LTG), AMPA receptor antagonist (perampanel; PER), SV2A modulator (levetiracetam; LEV), and α2δ-1 calcium channel subunit modulator (gabapentin; GBP).

### 2.6. Immunohistochemistry for cFos neuronal activation marker

Cohort #2 mice were stimulated at the same CC95 current used for the acute ASM testing. Mice were then left undisturbed for 90 min before being euthanized via live decapitation for the collection of brains directly into 4% paraformaldehyde (PFA); 24 h later, the brains were transferred into 30% sucrose solution in PBS for 48–72 h, flash frozen, and stored at −80°C until cryosectioning. Brains were sectioned between Bregma—AP: 1.58 and 2.38 using a Leica CM1860 cryostat at 20 μm onto charged superfrost slides (Fisher) for immunohistochemical processing.

The protein product of the immediate early-gene cFos was used as a marker of seizure-induced neuronal activation to identify the brain structures engaged by 6-Hz corneal stimulation (55), as previously published (44). After cryosectioning, slides were washed (3 × 5 min) in 0.1 M PBS. Slides were then permeabilized for 15 min with 0.2% Triton X-100 in 0.1 M PBS before being incubated in a 4% BSA blocking solution in 0.1 M PBS with 0.03% Triton X-100 under coverwells in a humid chamber for 2 h. The cFos antibody (1:1000; AB222699-1001, Abcam) was applied under 200 μL coverwells in a 1% BSA, 1% goat serum, and 0.03% Triton X-100 in 0.1 M PBS solution overnight at 4°C. The following day, coverwells were removed and slides washed in 0.1 M PBS (3 × 5 min) before being incubated with a goat anti-rabbit IgG H&L 555 nm secondary antibody (1:1000; AB150078 Abcam) in a 1% BSA, 1% goat serum, and 0.03% Triton X-100 in 0.1 M PBS solution light protected for 2 h at room temperature. The slides were again washed 3 × 5 min with 0.1 M PBS before being coverslipped with Prolong Gold with DAPI (ThermoFisher).

Photomicrographs were captured with a fluorescence microscope (Leica DM-4) with a 20x objective (80x final magnification) with acquisition settings held constant. cFos expression, given as average area, was automatically quantified as total field area with an immunofluorescent signal using Leica Thunder software. Additionally, the number of cells within each brain region that were positive for cFos labeling were hand-counted by two independent investigators blinded to the experimental group (Supplementary Figure 2), adapted from an ordinal scale similar to that previously reported (44). Brain regions assessed included subregions of the dorsal hippocampus, the posterior parietal association cortex overlaying CA1 of the dorsal hippocampus, and the piriform cortex.

### 2.7. Statistical analysis

The CC50s, CC95s, and ED50s were calculated by probit regression of binary data (56) using XLStat Life Sciences version 2019.1, or later with values confirmed to fall within the range of currents/doses tested. All binary response datasets for the ED50 calculations for male and female WT and PSEN2-KO mice are included in Supplementary Table 1. Statistical differences in ED50 or CC50 values were defined as values in which 95% confidence intervals did not overlap, consistent with probit methodology (56–58), which indicates with 95% probability that the true median value lies within this range. Importantly, confidence intervals provide an indication of the direction and strength of the effect studied and provide critical information about statistical differences between values that is more relevant than the *p*-value alone (59). All other statistical analyses were conducted in GraphPad Prism v8.0 or later, with *p* < 0.05 considered significant. Immunohistochemistry data for cFos labeling were checked for normality with a D'Agostino and Pearson test. A Mann–Whitney *U*-test was used to determine statistical differences in MMI following the administration of high-dose ASMs. Quantitative assessment of total area with cFos-positive signal was quantified with a two-way ANOVA with Sidak's *post-hoc* test.

## 3. Results

### 3.1. Valproic acid and levetiracetam are more potent in the 6-Hz seizure test in PSEN2-KO mice

**Figure 2:**
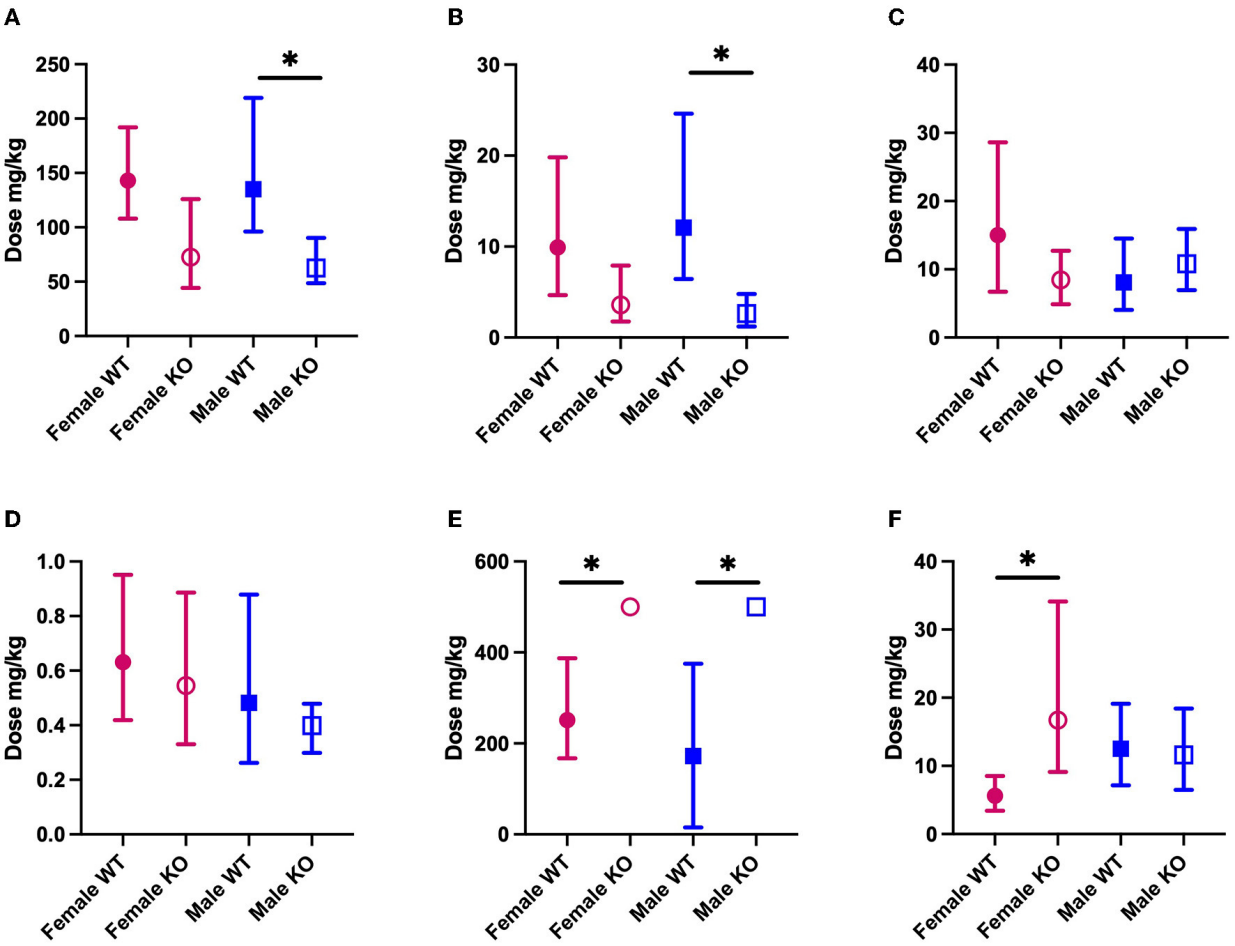
The median effective dose (ED50; the dose of an ASM that blocks seizures in 50% of animals tested) and 95% confidence intervals of each of the ASMs **(A)** VPA; **(B)** LEV; **(C)** LTG; **(D)** PER; **(E)** GBP; **(F)** CBZ in both male and female PSEN2-KO mice and their age-matched WT counterparts in the 6-Hz assay. Loss of normal PSEN2 function leads to significantly increased potency of VPA **(A)** and LEV **(B)** in male mice. There are no significant differences in the potency of LTG **(C)** of PER **(D)** between genotypes. Loss of normal PSEN2 function leads to significantly decreased potency of GBP **(E)** in male and female mice and of CBZ **(F)** in female mice. *Indicates non-overlapping 95% confidence intervals between WT and KO.

We sought to establish the ED50 of several mechanistically distinct ASMs in WT and PSEN2-KO mice to define the extent to which an AD-related genotype alone can influence the ASM activity profile in a well-characterized focal seizure model. There was some marked divergence in anticonvulsant activity in both male and female PSEN2-KO mice (Figure 2). The ED50 of VPA in male PSEN2-KO mice was 62.7 mg/kg [95% CI 48.6 – 90.4], which was significantly lower than the ED50 of VPA in male WT mice [135 mg/kg (96.2 – 219); Figure 2]. The female PSEN2-KO and WT mice followed a similar trend to the males, in that the VPA ED50 for PSEN2-KO females [72.7 mg/kg (44.3 – 126)] was lower than the VPA ED50 for WT females [143 mg/kg (108 – 192)], though this difference did not achieve statistical significance (Figure 2). Similarly, the ED50 of LEV in male PSEN2-KO mice [2.63 mg/kg (1.22 – 4.80)] was significantly reduced vs. that of male WT mice [12.1 mg/kg (6.44 – 24.6), Figure 2]. Females followed a similar trend, although the ED50s were not significantly different [PSEN2 KO: 3.59 mg/kg (1.76 – 7.90); WT: 9.92 mg/kg (4.66 – 19.8), Figure 2]. PSEN2-KO male mice were thus more sensitive to the broad-spectrum ASM, VPA, and the SV2A modulator, LEV, vs. WT in the 6-Hz assay. These data suggest that these ASMs, which act on glutamatergic synaptic vesicle release, were more potent in PSEN2-KO mice vs. age-matched WT mice.

### 3.2. There is no difference in the potency of lamotrigine and perampanel in PSEN2-KO mice in the 6-Hz seizure test

We sought to similarly establish the ED50 for both LTG and PER in PSEN2-KO mice as both ASMs are likely to be well-tolerated and used frequently in older adults with epilepsy (38, 53, 54). There was no difference in the ED50 of LTG in PSEN2-KO males [10.8 mg/kg (6.94 – 15.9)] relative to WT males [8.09 mg/kg (4.06 – 14.5)]. Furthermore, the ED50 of PER was not significantly different in PSEN2-KO males [0.399 mg/kg (0.299 – 0.479)] vs. WT males [0.482 mg/kg (0.262 – 0.879); Figure 2]. This effect was similarly evident in female PSEN2-KO mice; there was no difference between the female PSEN2-KO and WT mice ED50′s for LTG [PSEN2 KO: 8.45 mg/kg (4.89 – 12.7); WT: 15.0 mg/kg (6.73 – 28.6)]. There was also no difference in the potency of PER [PSEN2 KO: 0.545 mg/kg (0.330 – 0.886); WT: 0.631 mg/kg (0.419 – 0.951), Figure 2]. Thus, neither the sodium channel blocker, LTG, nor the AMPA receptor antagonist, PER, exhibited differences in antiseizure potency in PSEN2-KO mice relative to age- and sex-matched WT controls in the 6-Hz assay.

### 3.3. The potency of gabapentin in PSEN2-KO mice and of carbamazepine in female PSEN2-KO mice is reduced in the 6-Hz seizure test

There were two significant differences in ASM potency with GBP and CBZ, two agents frequently recommended for older adults with epilepsy (38, 53, 54), that largely work through fast neurotransmission via presynaptic ion channels (60). The ED50 of GBP was determined to be 172 mg/kg [15.4 – 375] for WT males and 251 mg/kg [167 – 387] for WT females (Figure 2). The ED50 for GBP could not be calculated for male or female PSEN2-KO mice (Figure 2), as only four of eight males and five of eight females were protected from a seizure at the highest dose tested (500 mg/kg, i.p.). At this same dose, six of seven WT males and seven of eight WT females were protected. Despite the inability to calculate an ED50 in PSEN2-KO mice, these results suggest that loss of normal PSEN2 function reduces the sensitivity to acute administration of the α2δ-1 calcium subunit channel modulator, GBP, in the 6-Hz assay. We also observed markedly reduced potency of CBZ in female PSEN2-KO mice. The ED50 of CBZ in female PSEN2-KO mice was 16.7 mg/kg [9.11 – 34.1] significantly higher than the CBZ ED50 in female WT mice [5.61 mg/kg (3.41 – 8.50), Figure 2]. Males were not significantly different [PSEN2 KO: 11.6 mg/kg (6.49 – 18.4); WT: 12.5 mg/kg (7.16 – 19.1), Figure 2]. Thus, female PSEN2-KO mice appear to be less sensitive to the acute administration of the sodium channel blocker CBZ compared with age-matched WT mice in the 6-Hz test; however, this trend was not conserved between the sexes. Altogether, these findings suggest that ASMs that exclusively target presynaptic ion channels necessary for fast neurotransmission may be less potent in PSEN2-KO mice in the 6-Hz limbic seizure test.

### 3.4. The protective index of selected ASMs is altered in PSEN2-KO mice

In addition to the assessment of anticonvulsant activity, mice were challenged on the rotarod immediately prior to seizure testing to determine the potential for MMI, consistent with routine ASM discovery practice (37, 41). While we did not determine a median motor-impairing dose (TD50) for any agent in this seizure model, the number of mice with motor impairment at the highest dose tested for each agent allowed us to estimate a relative PI for all compounds across the sexes and strains (Table 1) to directly compare with previously published values in other WT mouse strains (48, 61). There were no significant differences in MMI between PSEN2 KO and WT mice of either sex with the highest doses of GBP or LEV tested (Table 1). However, there were marked differences in tolerability for CBZ, PER, VPA, and LTG (Table 1). These findings altogether demonstrate that while some ASMs were not differentially potent in PSEN2-KO mice, there were marked and impactful differences in MMI and PI with these ASMs, which carries the potential to adversely affect tolerability in humans.

### 3.5. 6-Hz stimulation increases cFos protein expression in the posterior parietal association cortex and piriform cortex of WT mice, but not of PSEN2-KO mice

**Figure 3:**
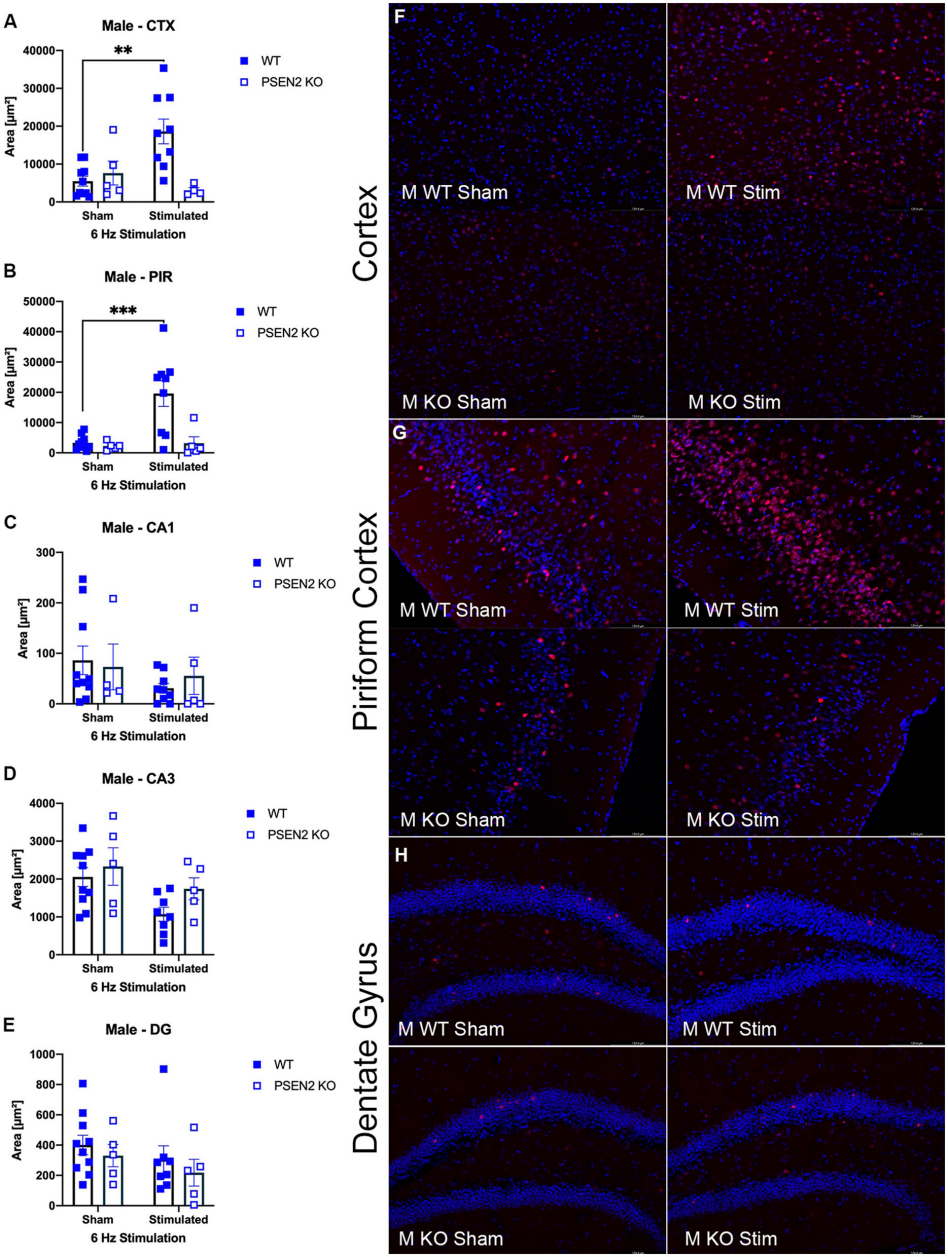
Immunohistochemical detection of the immediate early-gene cFos protein product was assessed in male wild-type (WT) and PSEN2-KO mice aged 3- to 4-month 90 min after a sham or single transcorneal 6-Hz stimulation. Brain regions analyzed for cFos expression by the automated Leica Thunder software include the following: **(A)** posterior parietal association cortex (region of cortex overlaying dorsal hippocampus at approximately Bregma −2.06); **(B)** piriform cortex; **(C)** area CA1 of the dorsal hippocampus; **(D)** area CA3 or dorsal hippocampus; **(E)** dentate gyrus (DG) of the dorsal hippocampus. The expression of cFos was analyzed by two-way ANOVA, and significance indicated within respective groups, where present (**indicates *p* < 0.01; ***indicates *p* < 0.001). Representative photomicrographs (80x final magnification) from regions where significant differences in cFos expression were appreciated in either males or females are included from **(F)** posterior parietal association cortex; **(G)** piriform cortex; and **(H)** dentate gyrus of the dorsal hippocampus. Representative photomicrographs of non-significant regions (CA1 and CA3) are included in Supplementary material.

**Figure 4:**
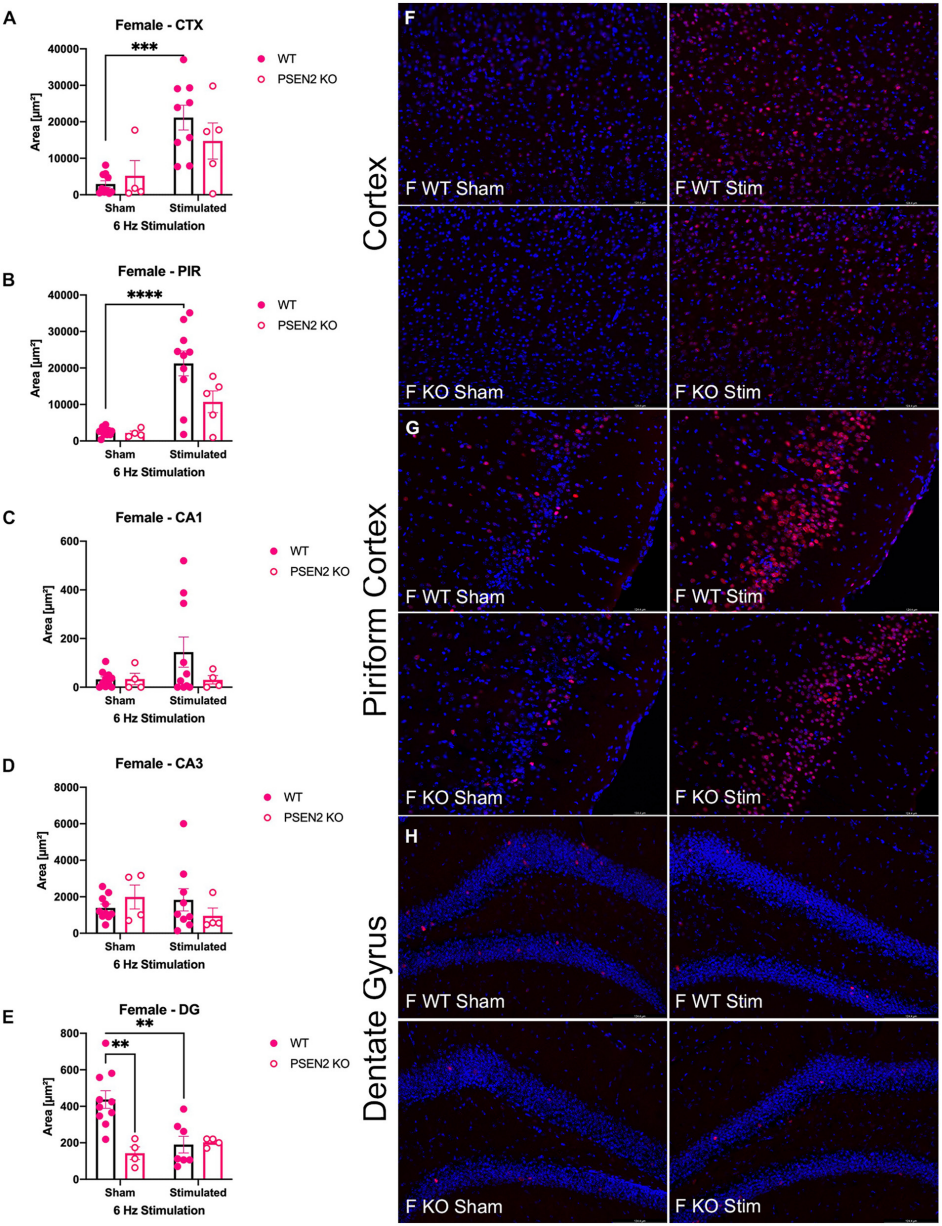
Immunohistochemical detection of the immediate early-gene cFos protein product was assessed in female wild-type (WT) and PSEN2-KO mice aged 3- to 4-month 90 min after sham or a single transcorneal 6-Hz stimulation. Brain regions analyzed for cFos expression by the automated Leica Thunder software include the following: **(A)** posterior parietal association cortex (region of cortex overlaying dorsal hippocampus at approximately Bregma −2.06); **(B)** piriform cortex; **(C)** area CA1 of the dorsal hippocampus; **(D)** area CA3 or dorsal hippocampus; **(E)** dentate gyrus (DG) of the dorsal hippocampus. The expression of cFos was analyzed by two-way ANOVA, and significance indicated within respective groups, where present (**indicates *p* < 0.01; ***indicates *p* < 0.001; ****indicates *p* < 0.0001). Representative photomicrographs (80x final magnification) from regions where significant differences in cFos expression were appreciated in either males or females are included from **(F)** posterior parietal association cortex; **(G)** piriform cortex; and **(H)** dentate gyrus of the dorsal hippocampus. Representative photomicrographs of non-significant regions (CA1 and CA3) are included in Supplementary material.

We performed immunohistological detection of the cFos protein product 90 min after a single 6-Hz stimulation to assess whether the observed differences in ASM potency or tolerability could be attributed to differences in regional activation in the brains of seizure-naïve male and female 3- to 4-month-old PSEN2-KO and WT mice. cFos is an immediate early gene that is activated and expressed in response to neuronal activity (55). The extent of cFos immunoreactivity was first qualitatively rated by two blinded, independent investigators to confirm 6-Hz stimulation-induced neuronal activation (Tables 2, 3). Expression of the protein product of cFos was then quantified for all mice in discrete brain regions (Figures 3, 4). There was notable upregulation of cFos expression in the brains of male WT mice, including a genotype x stimulation interaction on cFos expression in the posterior parietal association cortex [*F*_(1,24)_ = 10.08, *p* = 0.004; Figure 3A] and the piriform cortex [*F*_(1,25)_ = 6.649, *p* = 0.016; Figure 3B]. Only WT male mice demonstrated significant increases in cFos immunoreactivity in these regions in response to 6-Hz stimulation; PSEN2-KO male mice did not show similar neuronal activation in these regions.

**Table 2:** A single 6-Hz stimulation in male WT and PSEN2-KO mice aged 3–4 months induces qualitative regional differences in cFos expression, as evaluated by two independent investigators blinded to experimental conditions.

**Region**	**WT sham**	**WT stim**	**PSEN2-KO sham**	**PSEN2-KO stim**
CTX	3	3	3	3
PIR	3	4	2	3
CA1	1	1	1	0.5
CA3	2	1	2	2
DG	2	2	1	1

**Table 3:** A single 6-Hz stimulation in female WT and PSEN2-KO mice aged 3–4 months induces qualitative regional differences in cFos expression, as evaluated by two independent investigators blinded to experimental conditions.

**Region**	**WT sham**	**WT stim**	**PSEN2-KO sham**	**PSEN2-KO stim**
CTX	2	3.5	1.5	3
PIR	3	4	2.5	3
CA1	0.5	1	0.5	0.5
CA3	1	1	1	1
DG	2	1	1	2

The extent of cFos immunoreactivity in female mice subjected to 6-Hz stimulation (Figure 4) was also assessed, which expands on a study by Barton and colleagues who only assessed 6-Hz-induced cFos expression in male WT mice (44). There was a significant main effect of 6-Hz stimulation on cFos immunoreactivity in the posterior parietal association cortex [*F*_(1,24)_ = 17.35, *p* = 0.0003; Figure 4A] and the piriform cortex [*F*_(1,25)_ = 23.20, *p* < 0.0001; Figure 4B]. *Post-hoc* assessment in the posterior parietal association cortex revealed that cFos expression was only upregulated by 6-Hz stimulation in WT females (*p* = 0.0004); this assessment also showed only upregulated expression in the piriform cortex of WT females. In DG, there was a significant interaction on cFos expression in DG [*F*_(1,21)_ = 8.457, *p* = 0.0084; Figures 4E, H]. Although overall cFos immunoreactivity in the DG of female PSEN2-KO and WT mice was generally light (Figure 4H), *post-hoc* tests also revealed marked differences in stimulation-induced cFos expression in DG in WT females (*p* = 0.0031) that were not observed in PSEN2-KO mice. Thus, cFos immunoreactivity was significantly induced in the posterior parietal association cortex and piriform cortex of WT male and female mice, but this was not similarly observed in PSEN2-KO male and female mice. There were no major stimulation-induced changes in cFos immunoreactivity in the DG of PSEN2-KO mice, unlike effects observed in WT female mice. These findings suggest disrupted 6-Hz stimulation-induced brain region activation in PSEN2-KO mice relative to similarly stimulated WT counterparts.

## 4. Discussion

Seizures in people with AD are an emerging and untapped therapeutic opportunity to potentially alter the trajectory of the disease (6). These seizures also offer the opportunity to uncover potentially novel and biologically impactful, universally conserved mechanisms associated with seizures in older individuals (6, 11–13), which may benefit epilepsy patient populations more broadly (62). We have previously demonstrated that PSEN2-KO mice are useful to assess seizure susceptibility in an AD-associated genetic background (11), ASM response (11), and the impacts of chronic seizures on cognitive function (13). We herein demonstrate marked differences in ASM potency and tolerability in male and female PSEN2-KO mice vs. WT mice subjected to the 6-Hz model of evoked limbic seizures. We also demonstrate that loss of normal PSEN2 function may alter the PI of mechanistically distinct ASMs. While differences in mouse genetic strain can alone influence ASM potency (48), patterns of anticonvulsant activity are generally similar across strains, with differences also attributable to chemical source, formulation protocol, time of testing, route of administration, animal housing conditions, and animal age (44, 63–65). We herein demonstrated that age- and sex-matched PSEN2-KO mice exhibit notable sex- and strain-related differences in the patterns of ASM activity profiles relative to co-housed WT mice; findings suggest that loss of normal PSEN2 function disrupts ASM sensitivity beyond any variance attributable to genetic background strain alone. Furthermore, the WT mice in this study exhibited ED50 values and antiseizure activity patterns that are consistent relative to other WT strains (48, 63, 65). The ASMs VPA and LEV were substantially more potent in PSEN2-KO mice vs. age-matched WT animals. However, this change in potency was not universally observed with all ASMs tested. GBP was surprisingly ineffective in this seizure test in PSEN2-KO mice. CBZ demonstrated intriguing sex-related differences; it was more potent in female PSEN2-KO vs. WT mice, whereas it showed no differences in male PSEN2-KO vs. WT mice. Conversely, the potency of PER and LTG was unaltered in PSEN2-KO mice vs. WT mice. There were also substantial differences in the acute motor impairing effects of ASM administration in PSEN2-KO vs. WT mice (Table 1), suggesting that ASMs may be differentially tolerated in the setting of disrupted PSEN2 function. Finally, we demonstrate that 6-Hz stimulation in PSEN2-KO mice is associated with blunted cortical and piriform cortex activation, as assessed by cFos immunoreactivity. These findings cumulatively point to a substantial shift in ASM sensitivity and hyperexcitability in the context of loss of normal PSEN2 function.

While the PSEN2-KO mouse does not harbor a known EOAD PSEN2 gene variant (5, 25–27), clinical PSEN2 variants lead to a biochemical loss of normal function (66) such that PSEN-KO models are relevant to *a priori* assess the biological consequences of PSEN dysfunction in the setting of evoked secondarily generalized focal seizures. Despite the greater frequency of PSEN1 variants in EOAD, global PSEN1-KO mice are non-viable, whereas PSEN2-KO mice develop normally (49) and are viable up to at least 14 months old (13). Therefore, PSEN2-KO mice are useful to understand how global disruptions in PSEN signaling modify ASM activity profiles in the well-characterized evoked 6-Hz limbic seizure model, which is routinely used for frontline ASM discovery (35, 37, 41, 46, 47, 67). Notably, PSEN2-KO mice demonstrate high-frequency oscillations (68) and seizure-induced cognitive deficits (13), representing a suitably valid model of seizure-induced behavioral effects in an AD-associated genetic background. Until now, no study of ASM efficacy against evoked or spontaneous seizures has yet established a PI or defined the tolerability profile in a rodent AD-associated model, leaving a significant gap in knowledge with regard to the therapeutic window for the management of seizures in AD. Thus, our present study reveals likely mechanism-specific differences in ASM potency and acute tolerability in the AD-associated PSEN2-KO mouse that warrant more in-depth clinical study in genetically confirmed EOAD patients with seizures.

Limited prior clinical studies have investigated ASM use in older adults with mild-to-moderate AD and reported mixed therapeutic benefits. While VPA is generally acceptable for use in older adults with epilepsy (38, 53, 54), it may be contraindicated in patients with seizures in AD; a small study demonstrated that VPA administration led to increased brain volume loss and accelerated decline in MMSE scores (69, 70). Our present study revealed that MMI was worsened in PSEN2-KO male mice at the highest dose of VPA tested, but VPA was actually more potent against the 6-Hz secondarily generalized focal seizures in the PSEN2-KO mice at low doses. Our findings suggest that the therapeutic window of VPA may be shifted in this AD-associated model, which warrants further clinical study. The precise mechanism by which VPA exerts anticonvulsant effects is unclear, but it has been postulated to act through a diversity of molecular targets relevant to neuronal hyperexcitability (60) and AD (71, 72). While our current study was limited to the acute effects of VPA administration in relatively young animals, our findings of a shift in the PI of this agent suggest that perhaps the dose of VPA used in AD patients was higher than necessary to elicit neuroprotective and anticonvulsant benefits (69, 70) and thus resulted in the observed higher likelihood of treatment-related adverse side effects.

Chronic administration of the SV2A modulator, LEV, is both efficacious and well-tolerated in patients with mild-to-moderate AD (19, 73). Chronic administration of low-dose LEV may even improve performance on spatial memory and executive function tasks in patients with AD and epileptiform activity (22). In line with this clinical evidence, LEV did not elicit MMI in either genotype at the highest dose tested in our present study, and it potently blocked 6-Hz seizures at very low doses in the PSEN2-KO mice, suggesting a widened PI with this agent in PSEN2-KO mice. Our prior study with 60-Hz corneal-kindled PSEN2-KO mice also pointed to the increased potency of LEV in the absence of motor-impairing effects (11). Notably, this present study starkly contrasts with our earlier findings for reduced potency of LEV (and brivaracetam) in 6-Hz corneal-kindled mice in APP overexpressing AD models, revealing potential heterogeneity in ASM activity profiles in the setting of AD-related genotypes or intrinsic differences in the evoked seizure paradigms (12, 74). Thus, our current study suggests that the use of the acute 6-Hz limbic seizure model evoked in PSEN2-KO mice may beneficially identify both effective and well-tolerated agents for future clinical investigation to better therapeutically manage seizures in people with AD.

GBP is a calcium channel modulator that is a commonly prescribed ASM for older adults with epilepsy because of the minimal risk for drug–drug interactions and favorable cognitive side effect profile in this age group (38, 53, 54). However, studies of the safety and efficacy of GBP in older adults with seizures in AD are scant (75). GBP was entirely ineffective against 6-Hz focal seizures in PSEN2-KO mice in our study; whether PSEN2 or other AD-related variants are associated with altered GBP sensitivity requires further scrutiny as few preclinical studies have included this ASM for anticonvulsant testing in AD models. However, the PSEN2 protein is known to play a role in mitochondrial-dependent calcium homeostasis (76), which underlies normal neuronal signaling and seizures in epilepsy. Studies also suggest that EOAD-linked presenilin variants lower the calcium ion content of intracellular stores. By deleting the PSEN2 gene, it is likely that disrupted calcium homeostasis negatively influences the anticonvulsant potential of GBP, a calcium channel modulator, leading to our observed outcomes; a finding that warrants additional study.

While both LTG and CBZ are sodium channel blockers, the two ASMs illustrated very different anticonvulsant activity profiles likely owing to the additional effects of LTG on calcium channels (60). There were no differences in the potency of LTG between PSEN2-KO and WT mice of either sex. However, both male and female PSEN2-KO mice exhibited significant MMI at the highest dose of LTG tested relative to WT mice, revealing a narrower PI with LTG in PSEN2-KO mice. In contrast, both sexes of PSEN2-KO mice were less susceptible to the MMI-inducing effects of a high dose of CBZ compared with WT mice. PSEN2-KO females were also less sensitive to the anticonvulsant properties of CBZ than WT females in the 6-Hz test, reflective of a widened PI in this sex and strain. Thus, while the primary mechanism of action of these two ASMs is similar, differences exist in the metabolism and clearance of CBZ vs. LTG (77, 78), which may have also influenced our observed tolerability differences. Our present study did not evaluate plasma or brain concentrations of the selected ASMs; thus, future studies are needed to define the pharmacokinetic properties of candidate ASMs in this model and other AD-associated mouse models with evoked or spontaneous seizures to better establish the therapeutic potential of ASM use in people with AD.

AMPA receptor trafficking critically mediates normal synaptic plasticity and long-term potentiation (79, 80). In AD, AMPA receptor expression and trafficking are substantially dysregulated by the presence of amyloid β oligomers (81–84), thereby making modulation of AMPA receptors a relevant therapeutic target in AD. The non-competitive AMPA receptor antagonist PER has been shown to improve cognitive function and mediate psychiatric symptoms in an AD patient with myoclonic epilepsy due to its demonstrated anticonvulsant effects in this individual (85). Considering that PER is generally cognitively neutral in people with epilepsy (86), including in older adults with epilepsy (38), we sought to quantify the anticonvulsant potency and tolerability of PER against acute 6-Hz focal seizures in PSEN2-KO mice to further define therapeutic potential of this agent for older adults with seizures and AD. While the potency of PER in PSEN2-KO mice did not differ from WT animals, PER was much better tolerated in both male and female PSEN2-KO mice vs. their age-matched WT counterparts. Our results indicate a widened PI for PER in the PSEN2-KO genotype; mice were largely unimpaired by the highest dose of PER tested. These findings suggest that further detailed studies to assess the clinical benefit of PER use in older adults with seizures, including people with AD, are necessary. Given the distinct molecular anticonvulsant mechanism of PER coupled with the widened PI in our mouse model and the absence of cognitive liability in people with epilepsy, PER may be a reasonable therapeutic option to manage seizures in AD populations.

cFos is a useful marker of neuronal network activation following evoked seizures, including in response to a 6-Hz stimulation (44, 74). We observed that cFos was robustly upregulated in response to 6-Hz stimulation in the posterior parietal association cortex overlying dorsal hippocampus, as well as in the piriform cortex, in WT mice. There was blunted cFos expression in these regions in PSEN2-KO mice in response to this stimulation, despite the presentation of evoked seizures. These findings suggest that stimulation-induced neuronal network activation in PSEN2-KO mice is disrupted, in particular at the level of the cortex and piriform cortex. The piriform cortex is responsible for producing olfactory experiences (87) and memory encoding. It also frequently shows heavy cFos immunoreactivity in response to all 6-Hz stimulation currents (44). In fact, animal studies indicate that the piriform cortex is more prone to electrical stimulation-induced epileptic seizures than the hippocampus, amygdala, and entorhinal cortex (88). Moreover, evoked seizure activity tends to damage piriform cortex neurons (89, 90). While our current study was not designed to quantify longitudinal changes in piriform cortex size or volume, piriform cortex volume loss has been shown to be approximately twice as large as in the hippocampus in people with mild cognitive impairment (MCI) and AD (91), and also larger than the loss in the amygdala. Furthermore, piriform cortex atrophy is similarly apparent in patients with MCI as in those with AD, suggesting that piriform cortex atrophy may be a novel biomarker for early AD stages. Considering that we presently demonstrate reduced piriform cortex activation following a single 6-Hz electrical stimulation in mice with an AD-related genotype, further studies to assess the bidirectional relationship between seizures in AD and involvement of the piriform cortex are needed.

There is high translational value in this present study to improve ASM selection in patients with seizures in AD. The 6-Hz evoked seizure model is a well-characterized, frontline ASM discovery model that is routinely used to identify the anticonvulsant potential of novel therapeutic agents for epilepsy (37, 41, 44, 46, 47, 92). Importantly, the acute 6-Hz seizure model activates limbic structures known to be hyperexcitable in AD. However, aged rodents and rodent models with AD-related genotypes are infrequently integrated into initial ASM efficacy and tolerability studies (6, 37). Based on the bimodal age distribution of epilepsy prevalence being higher in the very young and the very old (38), it is necessary to more frequently include aged rodents or models of aging-related neurological disorders in ASM discovery practice (6, 93). Exclusively relying on efficacy studies in young, male, neurologically intact WT rodents will not adequately address the clinical needs of the world's rapidly increasing population of older adults (93). Consistent with efforts to increasingly integrate syndrome-specific models of rare pediatric epilepsies into routine ASM discovery practice (42, 43, 47), a strategy that also includes aging models or models of aging-related diseases into the ASM discovery pipeline could substantially benefit therapeutic innovation for older adults with seizures. Our present studies provide proof-of-concept demonstration that the use of mice with AD-associated genetic risk factors can uncover biologically relevant differences in ASM potency and tolerability. This study highlights a potential untapped opportunity to apply precision medicine strategies in the management of seizures in AD. Furthermore, the characterization of the ASM response of PSEN2-KO mice in the acute 6-Hz model closely aligns with NINDS Research Benchmarks for epilepsy to prioritize discovery for the many forms in which epilepsy presents clinically (39, 40). Thus, ASM screening in the acute 6-Hz model in PSEN2-KO mice addresses an urgent need to diversify preclinical research for ASM discovery so that therapeutic options for people with seizures in AD can be more rationally discovered and, ultimately, prescribed to minimize the burden of AD.

## Data availability statement

The raw data supporting the conclusions of this article will be made available by the authors, without undue reservation.

## Ethics statement

The animal study was reviewed and approved by University of Washington Institutional Animal Care and Use Committee.

## Author contributions

LL and MB-H contributed to the conception and design of the study, conducted the experiments, performed the statistical analysis, and contributed to the manuscript revision, read, and approved the submitted version. LL wrote the first draft of the manuscript. All authors contributed to the article and approved the submitted version.
