# Supplementary material for: Loss of normal Alzheimer's disease-associated Presenilin 2 function alters antiseizure medicine potency and tolerability in the 6-Hz focal seizure model
Source: Front Neurol. 2023 Aug 1;14:1223472. doi: 10.3389/fneur.2023.1223472 (PMC10427874; doi:10.3389/fneur.2023.1223472)
Supplement: Supplementary file 1 [file Table_1.PDF]

Supplemental Table 1. Raw binary seizure protection data for male and female PSEN2 KO and WT mice (aged 3-4-months old at time of testing) in the 6 Hz seizure test. Each ASM was tested at the predetermined time of peak effect (TPE). Data for each ASM in each sex and strain group are presented as: Dose (in mg/kg) - N/F (number protected/number tested). Median effective doses (ED50) for each ASM in each sex and strain were then calculated by a probit regression model (manuscript Figure 2).

ASM and Doses Tested (mg/kg, i.p.)	Time of Test (TPE), hr	M WT	M KO	F WT	F KO
VPA (300 mg/kg)	0.25	50 – 0/8; 75 – 3/8; 115 – 3/8; 150 – 4/8; 300 – 7/8	37.5 – 1/8; 50 – 2/7; 75 – 5/8; 150 – 7/7; 300 – 8/8	15 – 0/8; 37.5 – 2/8; 50 – 4/7; 75 – 6/8; 150 – 5/8; 300 – 8/8	50 – 0/8; 75 – 1/8; 115 – 4/8; 150 – 4/8; 225 – 6/8; 300 – 7/8
LEV (54 mg/kg)	1.0	1 – 0/8; 3 – 1/8; 7 – 3/8; 27 – 4/7; 54 – 8/8	0.5 – 0/6; 1 – 3/8; 3 – 4/8; 7 – 6/8; 14 – 6/7; 27 – 8/8	1 – 0/6; 3 – 2/8; 7 – 3/7; 14 – 5/8; 27 – 5/7; 54 – 7/8	0.5 – 0/8; 1 – 2/8; 3 – 4/8; 7 – 6/8; 14 – 6/8; 27 – 8/12; 54 – 7/8
LTG (56 mg/kg)	0.5	1 – 0/8; 3 – 2/8; 7 – 4/7; 14 – 6/8; 28 – 5/8; 56 – 8/8	3 – 0/8; 7 – 2/8; 14 – 6/8; 28 – 7/8; 56 – 8/8; 70 – 3/3	3 – 1/8; 7 – 3/8; 14 – 3/8; 28 – 6/8; 56 – 6/8; 70 – 7/8	3 – 1/8; 7 – 4/8; 14 – 7/12; 28 – 8/8; 56 – 8/8; 70 – 8/8
PER (2 mg/kg)	1.0	0.1 – 1/8; 0.25 – 2/8; 0.5 – 3/8; 1 – 7/8	0.25 – 0/8; 0.4 – 5/8; 0.5 – 6/8; 2 – 8/8	0.25 – 0/8; 0.4 – 1/8; 0.5 – 5/8; 1 – 8/8; 2 – 6/8; 3 – 7/8	0.1 – 0/8; 0.25 – 2/8; 0.5 – 3/8; 1 – 7/8; 2 – 7/8; 5 – 3/4
GBP (500 mg/kg)	2.0	10 – 2/8; 30 – 4/8; 75 – 3/8; 150 – 2/8; 300 – 6/8; 500 – 6/7	10 – 2/8; 30 – 5/8; 75 – 4/9; 150 – 4/8; 300 – 2/8; 500 – 4/8	10 – 1/8; 30 – 0/8; 75 – 3/8; 150 – 2/8; 300 – 5/8; 500 – 7/8	10 – 2/8; 30 – 5/8; 75 – 3/8; 150 – 1/8; 300 – 4/8; 500 – 5/8
CBZ (40 mg/kg)	0.25	5 – 2/8; 10 – 2/8; 20 – 5/8; 30 – 7/8; 40 – 6/6	1 – 0/7; 5 – 1/8; 10 – 3/8; 20 – 4/8; 30 – 7/8; 40 – 7/8	1 – 0/8; 3 – 2/8; 5 – 4/8; 10 – 6/8; 20 – 7/8; 30 – 5/5; 40 – 5/5	1 – 0/8; 5 – 2/8; 10 – 3/9; 20 – 5/9; 30 – 4/8; 40 – 7/8
