# Supplementary material for: Loss of normal Alzheimer's disease-associated Presenilin 2 function alters antiseizure medicine potency and tolerability in the 6-Hz focal seizure model
Source: Front Neurol. 2023 Aug 1;14:1223472. doi: 10.3389/fneur.2023.1223472 (PMC10427874; doi:10.3389/fneur.2023.1223472)

Supplemental Figure 1. Median convulsant current (CC50) curves were initially reproduced by an independent investigator (LL) to fall within previously published values (Beckman et al, *Neurobiol Dis* 2020) for both A) male and B) female PSEN2 KO mice aged 3-4 months old. CC50 values were determined using the Probit regression model applied to binary "yes/no" seizure presentation data from up to 8 mice per voltage step, with the current calculated through Ohm's law according to the resistance of the Grass S48 stimulation unit (2,672 ohm).

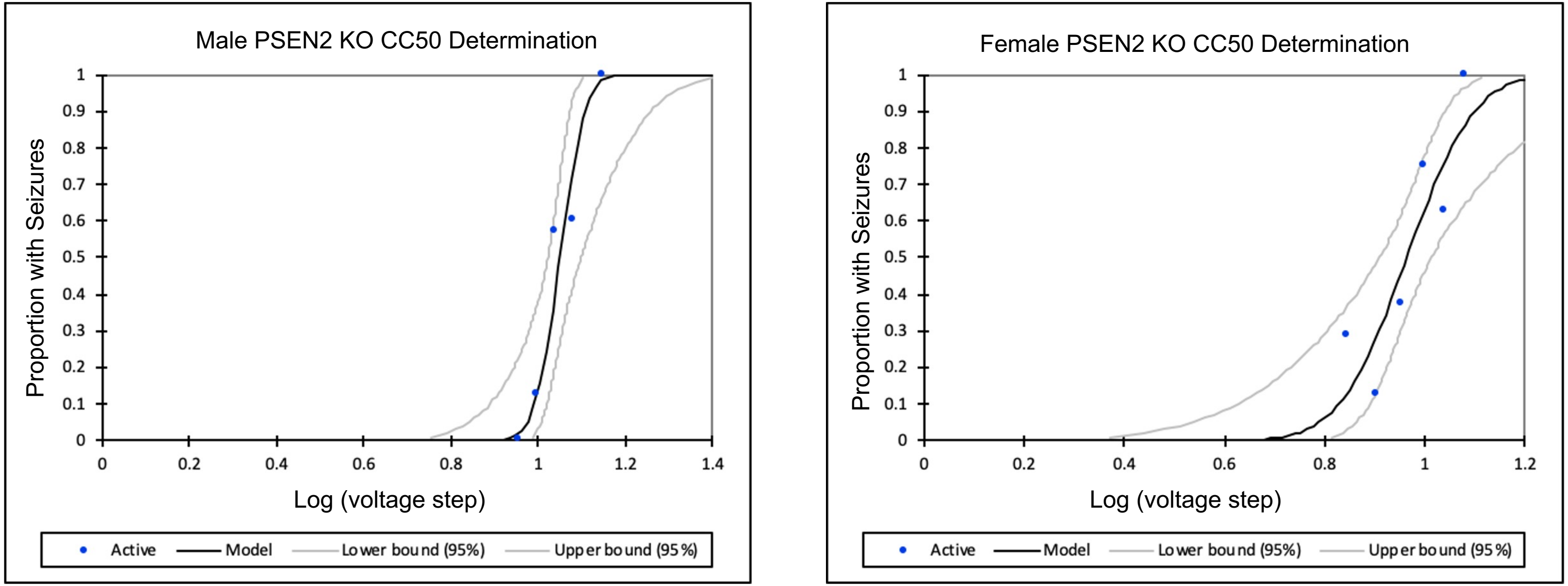

Supplemental Figure 2. An ordinal rating scale, similar to that which has been previously described by Barton and colleagues (Epilepsy Res 2001) was initially used by two independent investigators to confirm evidence of 6 Hz stimulation-induced expression of cFos protein product in neuronal circuits within hippocampal, cortical and piriform cortex structures. Representative images of scoring criteria are presented.

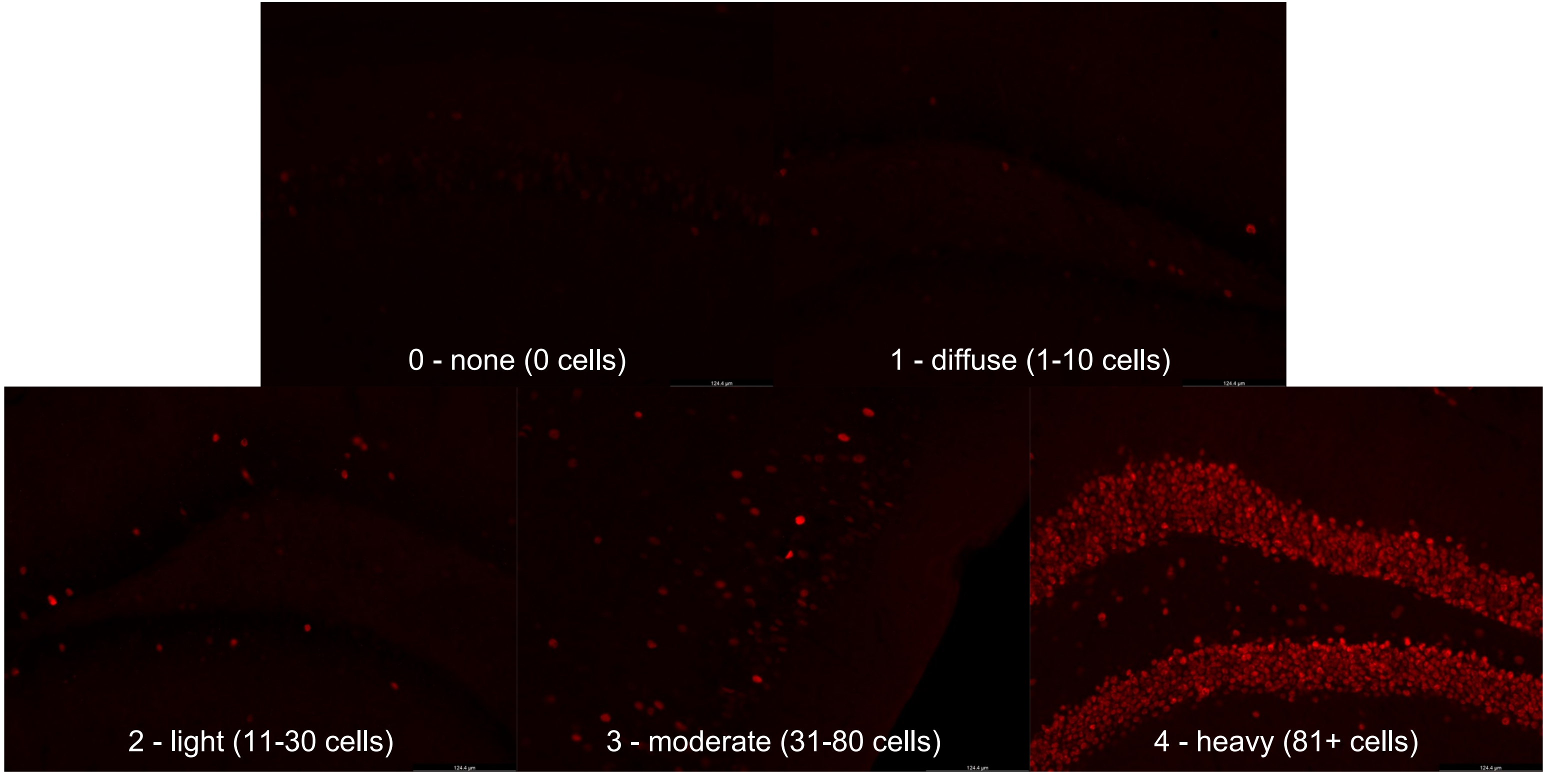

Supplemental Figure 3. Representative images of dorsal hippocampal brain regions A-D) CA1 and E-H) CA3 with non-significant changes in cFos immunoreactivity between male WT and PSEN2 KO mice aged 3-4 months-old following a single, transcorneal 6 Hz electrical stimulation. Red indicates cFos-positive cells, blue is the nuclear counterstain DAPI. Images are presented at 80x final magnification.

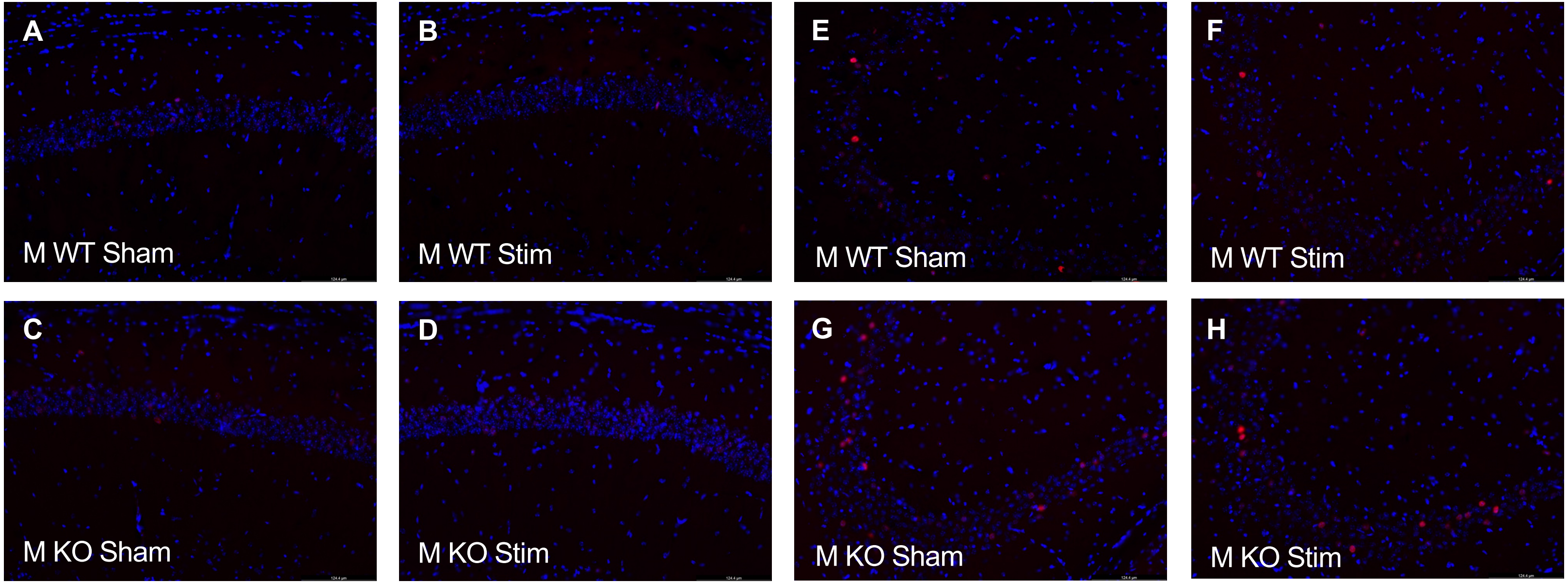

Supplemental Figure 4. Representative images of dorsal hippocampal brain regions A-D) CA1 and E-H) CA3 with non-significant changes in cFos immunoreactivity between female WT and PSEN2 KO mice aged 3-4 months-old following a single, transcorneal 6 Hz electrical stimulation. Red indicates cFos-positive cells, blue is the nuclear counterstain DAPI. Images are presented at 80x final magnification.

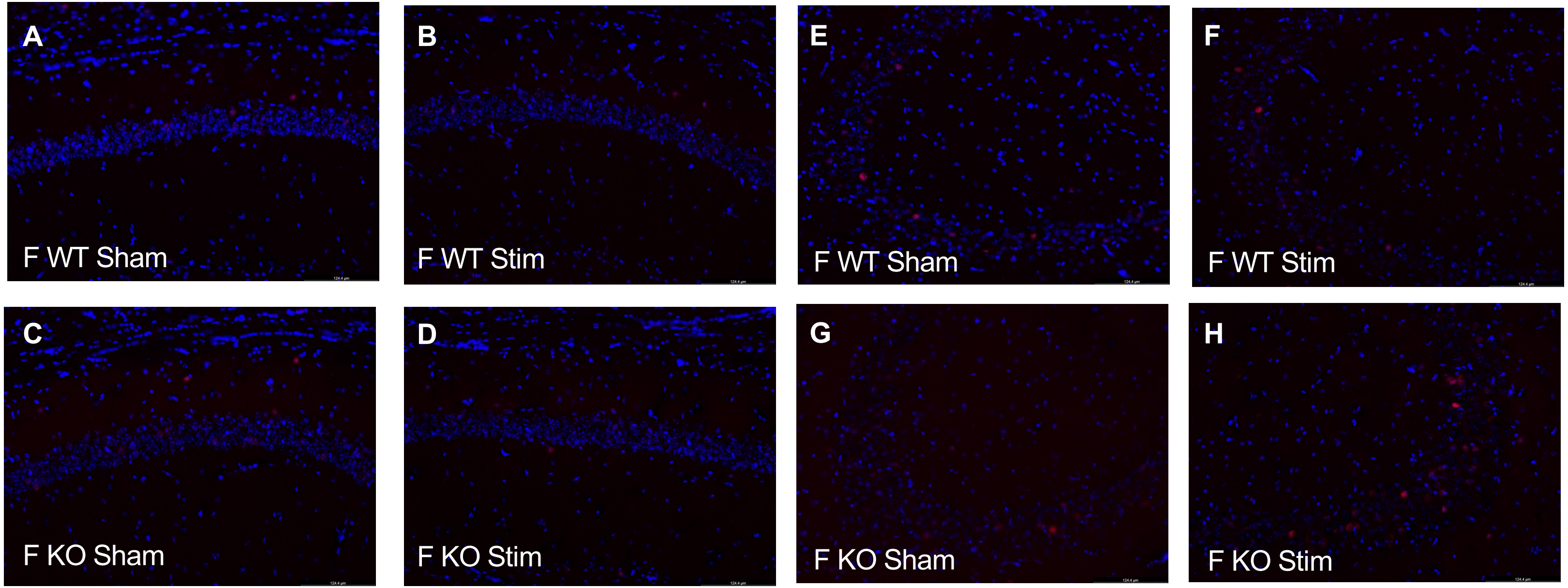

Supplement: Supplementary file 2 [file Data_Sheet_1.PDF]
